# Phenome-based gene discovery provides information about Parkinson’s disease drug targets

**DOI:** 10.1186/s12864-016-2820-1

**Published:** 2016-08-31

**Authors:** Yang Chen, Rong Xu

**Affiliations:** Department of Epidemiology and Biostatistics, Case Western Reserve University, Cleveland, OH USA

**Keywords:** Parkinson’s disease, Disease gene prediction, Network analysis, Drug discovery

## Abstract

**Background:**

Parkinson disease (PD) is a severe neurodegenerative disease without curative drugs. The highly complex and heterogeneous disease mechanisms are still unclear. Detecting novel PD associated genes not only contributes in revealing the disease pathogenesis, but also facilitates discovering new targets for drugs.

**Methods:**

We propose a phenome-based gene prediction strategy to identify disease-associated genes for PD. We integrated multiple disease phenotype networks, a gene functional relationship network, and known PD genes to predict novel candidate genes. Then we investigated the translational potential of the predicted genes in drug discovery.

**Results:**

In a cross validation analysis, the average rank for 15 known PD genes is within top 0.8 %. We also tested the algorithm with an independent validation set of 669 PD-associated genes detected by genome-wide association studies. The top ranked genes predicted by our approach are enriched for these validation genes. In addition, our approach prioritized the target genes for FDA-approved PD drugs and the drugs that have been tested for PD in clinical trials. Pathway analysis shows that the prioritized drug target genes are closely associated with PD pathogenesis. The result provides empirical evidence that our computational gene prediction approach identifies novel candidate genes for PD, and has the potential to lead to rapid drug discovery.

## Background

Parkinson’s disease (PD) is the second most common neurodegenerative disorder with a significantly increasing prevalence [1]. It involves pathological factors for cell death, such as mitochondrial dysfunction and oxidative stress [2, 3]. However, the highly complex and heterogeneous disease mechanisms are still inconclusive [4]. Current pharmacological treatment shows limited efficacy in reversing progressive neuronal loss and controlling nondopamineric symptoms, such as dementia and sensory disturbances [5, 6], which have become a major source of patient disability. Detecting novel genetic basis for PD not only reveals the disease pathogenesis, but also facilitates identifying novel drug targets [1–3, 7].

Overlapping disease phenotypes may indicate common genetic basis of the diseases [8]. Studying disease phenotypes of PD have the potential to uncover its underlying genetic factors [9, 10]. Previous studies have systematically analyzed disease networks based on phenotypic similarities to predict disease genes [11–14]. Currently, disease phenotype data sources remain largely incomplete. One disease phenotypic network is based on human phenotype ontology (HPO) [15] and has many applications [16–18]. Recently, we explored a new data source of human disease phenotype in biomedical ontologies and constructed the disease manifestation network (DMN). We showed that DMN contains new phenotypic knowledge and is useful in disease gene prediction [19]. In this study, we propose to combine DMN and HPO, and detect novel candidate disease-associated genes for PD using a network-based gene prediction strategy.

Several recent studies showed that matching the traits of genes in Online Mendelian Inheritance in Man (OMIM) [21] and genome-wide association study (GWAS) [22, 23] with the drug targets may lead to the discovery of new drug treatments. In a recent study, we proved that the disease-associated genes predicted by computational approaches also have the potential to guide drug discovery [24]. Here, we demonstrate that the candidate genes predicted for PD by our approach can provide information for PD drug targets. We evaluated the ranks of drug target genes for FDA-approved PD drugs and potential PD drugs that have been tested in clinical trials. We also performed pathway analysis for the top ranked drug target genes. The result provides empirical evidence that our gene prediction approach has the translation potential to lead to rapid drug discovery.

## Method

**Fig. 1:**
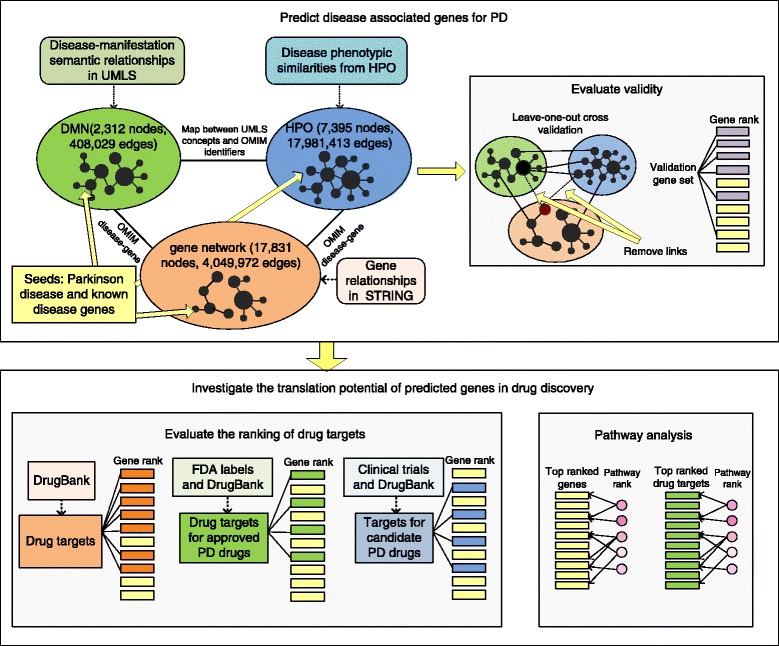
The study contains two steps: predict genes for PD and analyze the potential of predicted genes in drug discovery. We first predicted genes for PD from the integrated networks, and evaluated the prediction result. Then we assessed the whether the approach prioritizes drug target genes for PD

The work flow of our study is shown in Fig. 1 and consists of two parts: (1) predict genes for PD through network analysis and (2) investigate the translational potential of the predicted genes. In the first part, we combined the disease network of HPO, DMN, and a gene network, and used genes that are known to be associated with PD as the seeds to rank all the genes. The gene ranking result was validated in a “leave-one-out” cross validation and an experiment of prioritizing PD-associated genes obtained from GWAS. In the second part, we evaluated if the top-ranked genes are enriched for drug target genes for PD and provide opportunities for drug discovery.

### Predict genes for PD using a network-based approach

#### Construct networks

We downloaded the disease phenotype networks of HPO from http://human-phenotype-ontology.org and DMN from nlp.case.edu/public/data/DMN/. HPO contains 7395 nodes and 17,981,413 weighted edges. The disease phenotypic similarities are based on phenotype annotations extracted from OMIM, and were calculated as the semantic similarities in the phenotype ontology hierarchy [25]. DMN contains 2312 nodes and 408,029 weighted edges. The disease phenotype annotations were based on semantic network in the Unified Medical Language System (UMLS), and disease similarities were calculated as the cosine similarities between phenotype feature vectors between diseases [19]. Then we extracted 1,971,371 gene functional relationships from STRING [26] and constructed a gene network with 17,831 nodes. All data sources in STRING were used, including the protein interaction databases, pathway databases and gene coexpression data.

We constructed three bipartite networks to connect HPO, DMN and the gene network. We first extracted 4021 and 1872 disease-gene associations from OMIM to connect the disease nodes in HPO and DMN to the the gene nodes in the gene network, respectively. The disease nodes in HPO and DMN were represented by OMIM identifier and UMLS concept unique identifiers. Then, a total of 2250 maps between the two kinds of identifiers based on UMLS metathesaurus were used to connect HPO and DMN.

#### Predict candidate genes for PD

We first selected the seeds in the algorithm as the disease nodes of PD and their associated genes. PD has two forms: familial and sporadic. A major proportion of the patients have sporadic PD, and the associated genes in OMIM are for familial PD. However, extensive researches show that familial and sporadic PD are likely to share the same genetic pathways [27, 28]. Here, we extracted 15 PD genes from OMIM, and combined them with the PD disease nodes in both HPO and DMN to form a set of seeds.

Then we ranked all the gene nodes by their scores, which calculate the probabilities that each gene can be reached from the seeds. Assuming *p*_0_ is a vector of initial ranking scores, the updated score vector at step *k* is:

1 $$ {p_{k + 1}} = (1 - \gamma){M^{T}}{p_{k}} + \gamma {p_{0}},  $$

where *γ* is the probability that the random walker restarts from the seeds at each step, and *M* is the transition matrix of the entire heterogeneous network, which contains three intra-network transition matrices on the diagonal, and six inter-network transition matrices off-diagonal:

2 $$ \mathrm{M} = \left[ {\begin{array}{ccc} {{M_{G}}}&{{M_{G{P_{1}}}}}&{{M_{G{P_{2}}}}}\\ {M_{G{P_{1}}}^{T}}&{{M_{{P_{1}}}}}&{{M_{{P_{1}}{P_{2}}}}}\\ {M_{G{P_{2}}}^{T}}&{M_{{P_{1}}{P_{2}}}^{T}}&{{M_{{P_{2}}}}} \end{array}} \right].  $$

In the above equation, *P*_1_, *P*_2_ and *G* represent DMN, HPO and the genetic network, respectively. The diagonal sub-matrices *M*_*i*_(*i*∈*G*,*P*_1_,*P*_2_) were calculated through normalizing the adjacency matrix of *P*_1_, *P*_2_ and *G*, and the off-diagonal sub-matrices *M*_*ij*_(*i*,*j*∈*G*,*P*_1_,*P*_2_) were calculated through normalizing the bipartite network connecting *P*_1_, *P*_2_ and *G*. The normalization was performed following the method in [20].

#### Validate the gene prediction for PD

Before using this approach to predict new PD genes, we performed a cross validation analysis to test if the approach can identify the known disease-gene associations. For each of the 15 seed genes, we removed its connections to the PD nodes in HPO and DMN, and excluded it from the seed list. Then we used the rest seeds to rank all the genes. The procedure was repeated for 15 times, the ranks of the 15 genes were examined.

In the second validation experiment, we constructed an independent validation set containing 888 genes as a proxy of the novel PD genes. These genes were obtained through GWAS and downloaded from http://PDGene.org [29, 30]. We retained 669 genes, which have zero overlap with seeds and appear in our scope of gene ranking. We counted the number of validation genes in every 500 genes in our rank from the top to the bottom, and evaluated if the top ranked genes are enriched for the validation genes. We also generated the precision-recall curve to show the performance in ranking the validation genes.

### Evaluate the potential of the predicted genes in PD drug discovery

#### Investigate the ranks of drug target genes

Currently, only a subset of the human genome is druggable [31]. We investigated whether our approach can provide information about the drug target genes for PD. The ranking of two gene sets are tested: the first set contains target genes for FDA-approved PD drugs, and the second set contains target genes for potential PD drugs that have been tested in clinical trials. The drugs extracted from clinical trials are not necessarily successful PD therapies, but have been investigated by researchers for good reasons, thus are considered at least more promising than random drugs. We evaluate the ranking of target genes for both approved and potential PD drugs to approximate the ability of our approach in prioritizing PD drug targets. A total of 42 target genes for 22 FDA-approved PD drugs were extracted from DrugBank [32], which is a drug-target database. We also obtained 197 genes targeted by 81 PD drugs in http://clinicaltrials.gov (FDA-approved PD drugs were not included). Both sets of target genes have zero overlap with the seeds. We investigated their distributions among all genes.

#### Analyze pathways associated with top ranked genes

We included all the known PD-associated genes (including the genes identified by GWAS) into the seed list and predicted novel genes for PD. Then we analyzed the pathways associated with top-ranked candidate genes to detect their underlying commonalities. For each of the 1320 canonical pathways extracted from MSigDB [33], a score was calculated as the number of genes ranked within top 100 divided by the total number of genes in this pathway. The pathways with the highest scores offer insights into the functions of the predicted genes. In addition, we used the same method to analyze the pathways that are associated with the top 100 drug target genes.

## Results

### Network-based approach allowed prioritizing known PD-associated genes

In the leave-one-out cross validation, our approach prioritized the 15 known PD-associated genes from OMIM (the seed genes) in the top in each validation test. Table 1 shows that 13 out of 15 genes were ranked within top 1 %. A total of 12 genes were ranked within top 50 among all the 17,831 human genes. In all the 15 cases, the retained genes were ranked within top 10 %. The average rank for the retained seed genes is 147 (top 0.8 % among 17,831 genes).

**Table 1:** Result of the leave-one-out cross validation for 15 PD-associated genes from OMIM

Gene	Rank	Percentage
GBA	15	0.08 %
SNCA	17	0.10 %
MAPT	18	0.10 %
PLA2G6	20	0.11 %
TBP	23	0.13 %
HTRA2	23	0.13 %
PARK7	24	0.13 %
LRRK2	24	0.13 %
PARK2	24	0.13 %
PINK1	26	0.15 %
FBXO7	30	0.17 %
GIGYF2	33	0.19 %
SLC6A3	51	0.29 %
EIF4G1	361	2.02 %
VPS35	1521	8.53 %

We show the rank and percentage among all human genes

**Fig. 2:**
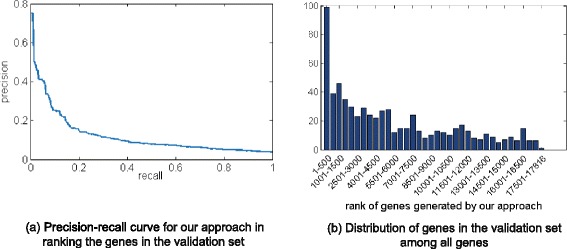
Distribution of genes in the validation set among all genes

In the second validation experiment, our approach prioritized the 669 validation genes, which are PD-associated genes detected by GWAS and related with different aspects of PD pathogenesis, such as mitochondrial dysfunction, oxidative stress and aging. Figure 2b shows the distribution of these genes among all.

The top 500 genes in the ranking contains 99 validation genes (5.3 fold-enrichment comparing with random rankings, *p*<*e*^−4^), and this number decreases rapidly as the rank changes from the top to the bottom. In Fig. 2a, the precision-recall curve also shows that the top-ranked genes are enriched for the PD genes detected by GWAS. The results demonstrate that the genes prioritized by our approach are likely to be associated with the pathogenesis of PD.

### Predicted genes have the translational potential in drug discovery

**Fig. 3:**
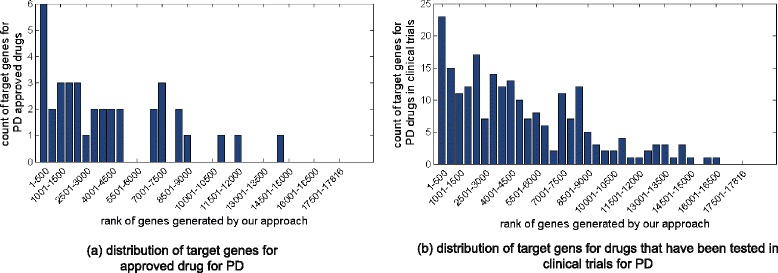
Distribution of target genes for approved PD drugs and candidate PD drugs that are tested in clinical trials

Figure 3 shows that our approach prioritized the genes targeted by FDA-approved PD drugs and potential PD drugs in clinical trials. The top 500 genes in the ranking contains 6 approved PD drug targets (including include COMT, DDC, DRD2, DRD3, HTR2A and MAOB), which is a 5.8-fold enrichment comparing with random rankings (*p*<*e*−4). Also, there are 23 potential PD drug targets in the top 500 genes (4.2-fold enrichment comparing with random cases, *p*<*e*−4). Figure 3a and b show the similar trends that the PD drug target genes are more likely to be ranked in the top than in the bottom. In addition, the top 500 genes contains 173 drug target genes, and 83 % of them have not been investigated for PD drug discovery. Together, these results suggest that the top-ranked candidate genes provides unique opportunities for detecting new candidate PD drugs through drug repositioning.

### Pathways underlying the top-ranked genes are associated with PD pathogenesis and provide information of potential PD treatments

The top ranked pathways associated with the newly predicted genes involve cell growth or degeneration, as listed in Table 2. Several among them are associated with nerve growth signalling (BIOCARTA_TRKA_PATHWAY) and aging (BIOCARTA_LONGEVITY_PATHWAY), which are closely related to neurodegenerative diseases and primary factors in the PD mechanism [1]. The result also shows that the top-ranked genes are associated with immunity, which is consistent with the literature evidence showing that immune responses can lead to the accumulation of neurotoxins and eventual neurodegeneration [34].

**Table 2:** Pathways that are enriched for the top ranked candidate genes for PD

Pathway	Description [33]
BIOCARTA_TRKA_PATHWAY	Nerve growth factor receptor signaling pathway
BIOCARTA_SPRY_PATHWAY	Regulation of cellular proliferation and differentiation
BIOCARTA_TFF_PATHWAY	Epithelial repair
BIOCARTA_ARF_PATHWAY	Tumor Suppressor that inhibits ribosomal biogenesis
BIOCARTA_LONGEVITY_PATHWAY	Involving age related diseases like neurodegenerative disease
BIOCARTA_NGF_PATHWAY	Nerve growth factor pathway
BIOCARTA_HER2_PATHWAY	Mediated signaling of EGFR
BIOCARTA_BCELLSURVIVAL_PATHWAY	Mediate the survival of B cells
BIOCARTA_CBL_PATHWAY	Downregulate EGF receptors
BIOCARTA_CTCF_PATHWAY	Induction of cell cycle arrest and apoptosis

We also ranked the pathways associated with the top drug targets. Table 3 shows the top ten pathways. Besides the same pathways involving nerve growth as in Table 2, the drug target genes are also linked to other genetic factors, such as the insulin-like growth factor and the active protein that controls cellular processes. The top one pathway BIOCARTA_IGF1_PATHWAY involves the insulin-like growth factor 1 (IGF-1) signaling. Previous researches support that IGF-1 has the potential to become a neuroprotective agent for PD. Animal model studies have demonstrated that IGF-1 provides protection against loss of dopaminergic neurons [35]. Several sequential studies also found that serum IGF-1 is increased in early idiopathic PD patients [36, 37].

**Table 3:** Pathways that are enriched for the top ranked drug target genes

Pathway	Description [33]
BIOCARTA_IGF1_PATHWAY	Stimulates cell growth and blocks apoptosis
BIOCARTA_INSULIN_PATHWAY	Regulation of glucose levels
BIOCARTA_NGF_PATHWAY	Nerve growth factor pathway
BIOCARTA_TRKA_PATHWAY	Nerve growth factor receptor signaling pathway
REACTOME_ACTIVATION_OF_THE_AP1	Activation of the AP-1 family of transcription factors
_FAMILY_OF_TRANSCRIPTION_FACTORS	
BIOCARTA_TFF_PATHWAY	Epithelial repair
BIOCARTA_LONGEVITY_PATHWAY	Involving age related diseases
REACTOME_SHC1_EVENTS_IN_EGFR	
_SIGNALING	EGFR signaling
BIOCARTA_CBL_PATHWAY	Downregulate EGF receptors
BIOCARTA_CDK5_PATHWAY	Cellular proliferation and survival

In summary, the pathway analysis detected the commonalities underlying the predicted PD genes. The prioritized pathways not only reflect PD genetic mechanisms, but also may lead to the discovery of targets for novel PD drug therapies.

## Discussion and conclusions

In this study, we propose a disease gene discovery strategy for PD, which integrates multiple disease phenotypic networks with gene functional relationships and known disease-gene associations. We validated our gene ranking with a cross validation analysis and an independent validation set. We demonstrated that the gene prediction approach provides information for the PD drug targets. The top ranked genes are enriched for targets for both approved and potential PD drugs, and provide unique opportunities for PD drug discovery.

Our approach can be further improved as more human disease phenotype data become available. For example, other kinds of disease phenotype data, such as disease co-morbidities [38, 39] and gene expression profiles, may reflect different aspects of genetic mechanisms and lead to the identification of novel candidate drug targets for PD. In the future, we will develop new approaches to rationally integrate heterogeneous human phenotype data.

In addition, we will systematically predict candidate drugs for PD using the gene prioritization result. Many existing drug discovery approaches compare the genetic and genomic features between diseases and drugs to identify candidate drug therapies [40]. Recent studies show that the phenotypic annotations for mouse gene mutations provide causal relationships between genes and phenotypes, and have great potential in drug repositioning [41, 42]. In our previous work, we designed a drug repositioning approach to combine the human disease genetics with the mouse phenotype data, and predict drugs for a given disease through comparing the phenotype profiles [43]. In the furture, we will incorporate the result obtained in this study into the drug repositioning approach, and improved the approach by combining other data, such as the drug actions and drug structural similarity.

In this study, we evaluated the ranking of genes and drug targets that are known to be associated with PD to approximate the performance of the computational disease-associated gene prediction approach. The ultimate goal of this approach is to identify novel genes and drug targets for PD. In the future, we plan to validate the newly predicted disease-associated genes and candidate drug targets through collaborative biomedical experiments and animal model studies.

## Abbreviations

DMN, disease manifestation network; FDA, Food and Drug Administration; GWAS, genome-wide association study; HPO, human phenotype ontology; IGF-1, insulin-like growth factor 1; OMIM, Online Mendelian Inheritance in Man; PD, Parkinson’s disease; UMLS, Unified Medical Language System
